# Identification and validation of a risk model and molecular subtypes based on tryptophan metabolism-related genes to predict the clinical prognosis and tumor immune microenvironment in lower-grade glioma

**DOI:** 10.3389/fncel.2023.1146686

**Published:** 2023-02-28

**Authors:** Wenxia Li, Ling Ling, Lei Xiang, Peng Ding, Wei Yue

**Affiliations:** ^1^Clinical College of Neurology, Neurosurgery and Neurorehabilitation, Tianjin Medical University, Tianjin, China; ^2^Department of Neurology, Tianjin Huanhu Hospital, Tianjin, China

**Keywords:** tryptophan, metabolism, lower-grade glioma, prognosis, tumor immune microenvironment

## Abstract

**Background:**

Lower-grade glioma (LGG) is one of the most common malignant tumors in the central nervous system (CNS). Accumulating evidence have demonstrated that tryptophan metabolism is significant in tumor. Therefore, this study aims to comprehensively clarify the relationship between tryptophan metabolism-related genes (TRGs) and LGGs.

**Methods:**

The expression level of TRGs in LGG and normal tissues was first analyzed. Next, the key TRGs with prognostic value and differential expression in LGGs were identified using the least absolute shrinkage and selection operator (LASSO) regression analysis. Subsequently, a risk model was constructed and Consensus clustering analysis was conducted based on the expression level of key TRGs. Then, the prognostic value, clinicopathological factors, and tumor immune microenvironment (TIME) characteristics between different risk groups and molecular subtypes were analyzed. Finally, the expression, prognosis, and TIME of each key TRGs were analyzed separately in LGG patients.

**Results:**

A total of 510 patients with LGG from The Cancer Genome Atlas (TCGA) dataset and 1,152 normal tissues from the Genotype-Tissue Expression (GTEx) dataset were included to evaluate the expression level of TRGs. After LASSO regression analysis, we identified six key TRGs and constructed a TRGs risk model. The survival analysis revealed that the risk model was the independent predictor in LGG patients. And the nomogram containing risk scores and independent clinicopathological factors could accurately predict the prognosis of LGG patients. In addition, the results of the Consensus cluster analysis based on the expression of the six TRGs showed that it could classify the LGG patients into two distinct clusters, with significant differences in prognosis, clinicopathological factors and TIME between these two clusters. Finally, we validated the expression, prognosis and immune infiltration of six key TRGs in patients with LGG.

**Conclusion:**

This study demonstrated that tryptophan metabolism plays an important role in the progression of LGG. In addition, the risk model and the molecular subtypes we constructed not only could be used as an indicator to predict the prognosis of LGG patients but also were closely related to the clinicopathological factors and TIME of LGG patients. Overall, our study provides theoretical support for the ultimate realization of precision treatment for patients with LGG.

## 1. Introduction

Glioma, which deriving from the neuroepithelial tissue, is one of the most common primary malignant tumors in the central nervous system (CNS) (Yang et al., 2022). Gliomas are responsible for approximately 30% of CNS tumors and 80% of malignant intracranial tumors (Ostrom et al., 2022), with a high incidence and mortality rate (Siegel et al., 2022). According to the World Health Organization (WHO) classification system in 2021 for CNS tumors, gliomas are classified as WHO grade I-IV. The lower-grade gliomas (LGGs), WHO grade II and III, mainly include three subtypes with histological and molecular characteristics: IDH mutant diffuse astrocytomas, IDH wild type diffuse astrocytomas and IDH mutations combined with 1p/19q codeletions in oligodendrogliomas (Louis et al., 2016). LGG exhibits significant intrinsic heterogeneity in its clinical and histological features (Aoki et al., 2018), with some patients expressing a long time progression-free survival (Brat et al., 2015), while others still having a certain recurrence and malignant transformation rates with poor survival prognosis, could rapidly developing into the highly aggressive secondary glioblastoma after standardized treatments (Xu et al., 2020). Since the uncertainty of LGG prognosis poses a challenge to clinical management, there is an urgent need for finding novel biomarkers to establish molecular subtypes and predict the prognosis of LGG patients, in order to support the more precise and individualized treatment.

Metabolism plays an important role in life activities and disease development. Compared with normal tissues, tumor tissues have significant metabolic abnormalities. Tumor cells can maintain their proliferation and progression by altering their metabolic patterns to obtain essential nutrients from a nutrient-deficient environment and change the tumor immune microenvironment (TIME) (Warburg, 1956). Therefore, as one of the hallmark features of tumors, metabolic reprogramming has an essential function in the pathogenesis of tumors, providing the necessary fundamental substance for the tumor cells and contributing to the biological behavior of tumors (Vaupel et al., 2019). In mammals, tryptophan, an essential amino acid, is associated with a variety of biological processes. In recent years, the relationship between tryptophan metabolism and tumors has been the focus of the research. A growing evidence suggests that tryptophan metabolism is involved in the development of tumors *via* multiple mechanisms. For example, Bosnyák et al. (2016) reported the characteristics of tryptophan metabolism in glioma patients and found that their oligodendrocytes and neurons were able to intake tryptophan and that the levels of the tryptophan catabolism production quinolinic acid in the cerebrospinal fluid were increased, suggesting that tryptophan metabolism is engaged in the pathophysiological process of glioma. Study have found that the expression of the speed-limiting enzyme in tryptophan catabolism process, tryptophan 2,3-dioxygenase is upregulated in glioma and other types of cancer, thus mediating the immune escape mechanism of tumors (Prendergast et al., 2018). In addition, studies have shown that Interleukin 4-inducible-1 (IL4I1), another enzyme in the tryptophan metabolism, is negatively correlated with the overall survival (OS) of glioma patients (Sadik et al., 2020). Nevertheless, there is still a paucity of studies on the role of tryptophan metabolism-related genes (TRGs) in LGG patients.

Therefore, this study extracted RNA sequencing data and clinical characteristics of LGG patients from public databases and intended to explore the expression pattern and prognostic value of TRGs in LGG patients. And we also explored the correlation between TRGs and clinicopathological factors and TIME of LGG patients. Furthermore, we developed a risk model and molecular subtypes by using the expression level of key TRGs to more precisely predict the prognosis of LGG patients. Briefly, in this study, we attempted to illuminate the clinical value of TRGs in LGG patients using bioinformatics analysis and determined the important role of tryptophan metabolism in LGG metabolic reprogramming, in order to offer the theoretical support for more precise and individualized treatment for LGG patients.

## 2. Materials and methods

### 2.1. Acquisition and preprocessing of LGG dataset and tryptophan-related genes

The transcriptome data of 510 patients with LGG and 1,152 normal tissues were downloaded from the Cancer Genome Atlas (TCGA) dataset (Wang et al., 2016) (TCGA-LGG)^1^ and the Genotype-Tissue Expression (GTEx) dataset (Carithers et al., 2015)^2^, respectively. After eliminating batch effects, a total of 1,662 tissues were regarded as the training cohort. Furthermore, we used the Chinese Glioma Genome Atlas (CGGA) dataset (Zhao et al., 2021)^3^ including 273 LGG patients as the validation cohort. The patients with grade II and III glioma and complete survival information were enrolled in this research. In this study, the TRGs were obtained from the MSigDB database (Liberzon et al., 2015)^4^ including KEGG_TRYPTOPHAN_METABOLISM, REACTOME_TRYPTOPHAN_CATABOLISM, and WP_TRY PTOPHAN_METABOLISM. After removing the duplicate genes, a total of 50 TRGs were enrolled in this study (Supplementary Table 1).

### 2.2. Identification of key tryptophan-related genes

Firstly, we analyzed the expression patterns of 50 TRGs in 510 LGG patients and 1,152 normal tissues from the training cohort. In this study, TRGs with *P*-value < 0.05 were considered as the differential expressed TRGs (DE-TRGs). Next, we further evaluated the prognostic value of 50 TRGs in 510 LGG patients from the TCGA-LGG dataset using “survival” package to perform univariate Cox regression. And a *P* < 0.05 was considered statistically significant. Subsequently, the Venn diagram was utilized to identify the overlapped TRGs between DE-TRGs and prognostic TRGs. Finally, the least absolute shrinkage and selection operator (LASSO) regression analysis was conducted for selecting and identifying the key TRGs associated with the OS rate *via* the “glmnet and survival” package in R software.

### 2.3. Construction of a prognostic risk model based on the key TRGs

On account of the risk coefficients calculated from the LASSO regression analysis, we established a risk model based on the key TRGs and calculated the risk score for each LGG patient from the TCGA-LGG dataset. Score = Coef-Gene_1_ × Exp-Gene_1_ + Coef-Gene_2_ × Exp-Gene_2_ + Coef-Gene_3_ × Exp-Gene_3_ + …… + Coef-Gene_N_ × Exp-Gene_N_, in which Coef-Gene_N_ represents the regression coefficient of Gene_N_ and Exp-Gene_N_ represents the expression level of Gene_N_. According to the median risk score, 510 LGG patients from the TCGA-LGG dataset were classified into different clinical groups: low- and high-risk groups. The Kaplan-Meier survival curve and the Log-Rank algorithm were performed to compare the OS of LGG patients between the low- and high-risk groups. And the time-dependent receiver operating characteristic (ROC) curves were used to calculate the area under curve (AUC) values to assess the prognostic predictive performance of the risk model *via* “survival ROC” in R software. In addition, we compare the AUC values of our risk model with those of similar previous models from other studies (Lai et al., 2022a; Zhang et al., 2022; Zhao et al., 2022) to further validate the predictive performance of the risk model.

### 2.4. Consensus clustering analysis and molecular subtypes construction

In order to determine whether TRGs could classify LGG patients, we carried out unsupervised K-means clustering analysis. The “ConsensusClusterPlus” package in R software was utilized with 1,000 times verifications to conduct the consensus clustering analysis for subtyping the LGG patients from the TCGA-LGG dataset according to the expression values of the key TRGs in each sample of the TCGA-LGG dataset. We confirmed the number of clusters and divided the LGG patients into different molecular subtypes according to the criteria: the cumulative distribution function (CDF) curve showed a smooth raising; the intra-cluster correlation was highest and inter-group correlation was lowest after clustering. Principal component analysis (PCA) was carried out to check the accuracy of the molecular subtypes *via* the “ggplot2” package in R software. At the same time, the Kaplan-Meier survival curve and the Log-Rank algorithm were performed to evaluate the prognosis between the two clusters. In addition, we performed gene set enrichment analysis (GSEA) with “c2.cp.all.v2022.1.Hs.symbols.gmt” from the molecular signatures database to focus on differential aggregation of molecular pathways between different clusters. A *P*-value < 0.05 was selected as differential aggregation of pathways.

### 2.5. Clinical and prognostic analysis of risk model and molecular subtypes

The primary endpoint of this study was OS in patients with LGG. Univariate and multivariate Cox regression analyses were used to investigate the association of age, gender, histological grade, 1p/19q codeletion, IDH1 status, risk score, and molecular subtype with OS to identify independent predictors of OS in LGG patients. To enhance the clinical applicability, we constructed the nomograms of 1-, 3-, and 5-year OS of LGG patients combined the independent clinical predictors (including age, histological grade, and IDH1 status) and risk score or molecular subtype from the results of multivariate Cox regression analysis. After 1,000 bootstrap resamples, we calculated the C-index and plotted the calibration curves to evaluate the predictive performance and robustness of the nomograms.

Furthermore, based on the clinicopathological factors of LGG patients from the TCGA-LGG dataset, we explored the relationship between risk scores or clusters and age, gender, histological type, histological grade, 1p/19q codeletion, and IDH1 status of LGG patients to further determine the clinical value of the risk model and molecular subtype.

### 2.6. Tumor immune characteristics analysis

The “ESTIMATE” package in R software was used to calculate the stromal score, immune score, ESTIMATE score, and tumor purity of each LGG samples. Violin plots were used to show the differences between different groups. Subsequently, we explored the levels of tumor microenvironment scores in different risk groups or clusters. In addition, the relative infiltration abundance of 22 immune cells among each LGG samples were calculated based *via* “CIBERSORT” algorithm. In each sample, the total of all estimated immune cell type scores equals 1. Meanwhile, we compared the relative infiltration abundance of immune cells in different risk groups or clusters using spearman rank correlation analysis.

### 2.7. External validation of the TRGs prognostic risk model and molecular subtypes

In this analysis, 273 LGG patients from the CGGA dataset were used as the external independent validation cohort to verify the risk model and molecular subtype. According to the formula developed in the training cohort, the risk score was calculated for each LGG patient in the validation cohort. And we used the Kaplan-Meier survival curves and the Log-Rank test to assess whether there was a significant difference in OS between the low- and high-risk groups. Besides, the prognostic prediction of the risk model was validated using the time-dependent ROC curves to calculate the 1-, 3-, and 5-year AUC values in the validation cohort. Finally, we verified the molecular subtypes and the prognostic value of two distinct clusters based on the expression level of key TRGs in the validation cohort.

### 2.8. Expression, prognostic value, and TIME analysis of key TRGs

After searching the Human Protein Atlas (HPA) database (Thul et al., 2017)^5^, we acquired the typical immunohistochemical staining images to visualize the expression level of TRGs in LGG tissues and normal tissues. Similarly, the prognostic value of the above key genes was explored in the validation cohort using the “survminer” R package. Spearman correlation analysis was utilized to explore the correlations between the key TRGs and the tumor microenvironment scores and 22 immune cells.

### 2.9. Statistical analysis

We used the Shapiro-Wilk normality test to assess whether the samples obeyed a normal distribution. For samples that did not satisfy the normal distribution, the Wilcoxon rank sum test was used to compare the differences between the two groups. The Kruskal-Wallis *H*-test, the Dunn’s test, and the Wilcoxon rank sum test were performed to evaluate the differences in clinicopathological features between the low-risk and high-risk groups. In addition, the Chi-square test was used to explore the differences in clinicopathological features between two molecular subtypes. The Kaplan-Meier survival curve and the Log-Rank test were used to perform the survival analysis of LGG patients. Multivariate Cox regression analysis was applied to identify the independent predictors of OS in LGG patients. Spearman correlation were utilized to explore correlation coefficients. We used R software (version 3.6.3) with its support packages to conduct the statistical analysis and to plot the figures. A *P*-value < 0.05 was considered significant statistically.

## 3. Results

### 3.1. Construction of a risk model based on TRGs in LGG

#### 3.1.1. TRGs expression patterns and the construction of a prognostic risk model in LGG

**FIGURE 1:**
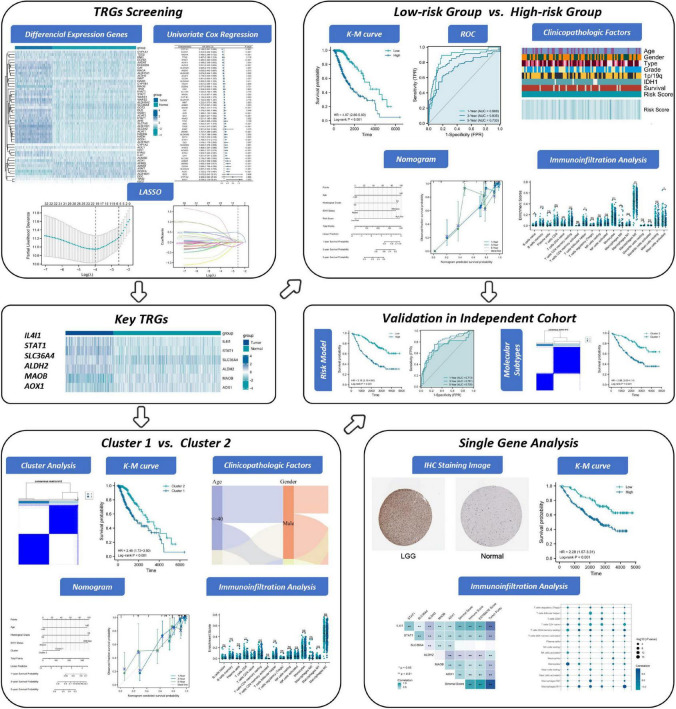
The flowchart of this study.

**FIGURE 2:**
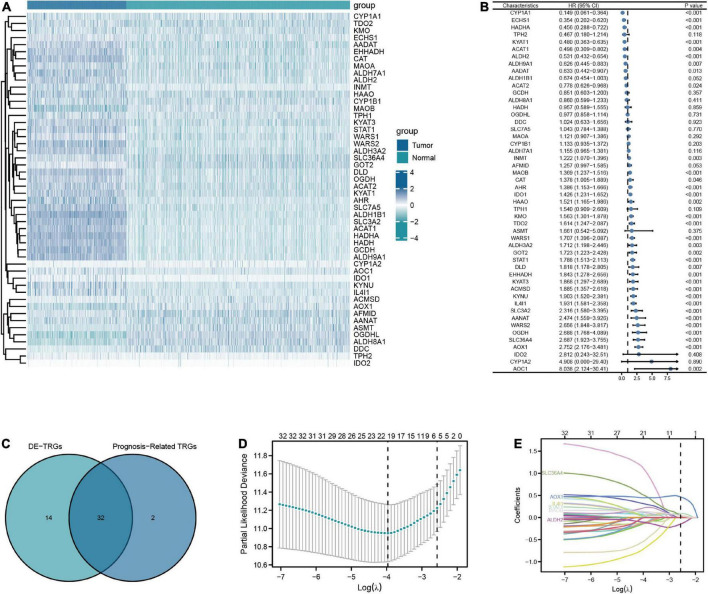
Screening for key TRGs in LGG patients. **(A)** The expression level of 50 TRGs between patients with LGG from TCGA-LGG dataset and normal tissues from GTEx dataset. **(B)** Forest plots of univariate Cox regression analysis of 50 TRGs in LGG patients from TCGA-LGG dataset. **(C)** The Venn diagram of overlapping between DE-TRGs and prognosis-related TRGs. **(D,E)** The LASSO regularized Cox regression analysis of key TRGs.

**FIGURE 3:**
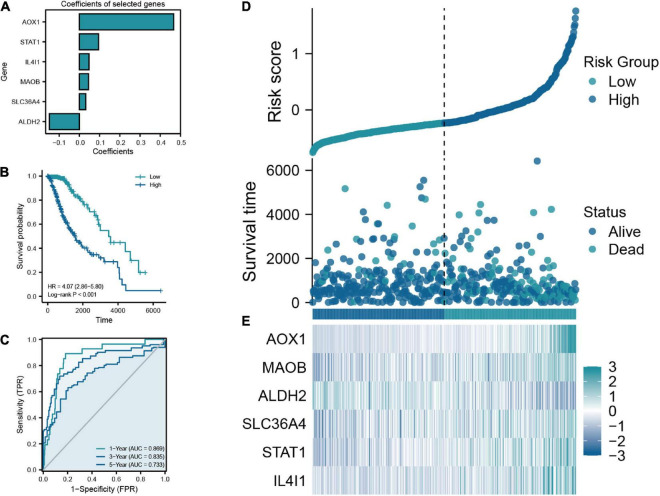
**(A)** Coefficients of the six selected genes and the risk score formula. **(B)** The Kaplan-Meier plot comparing the low- and high-risk groups in overall survival (OS). **(C)** The ROC curve for low- and high-risk groups in the train cohort. **(D)** Scatter diagram of risk score and survival status in the train cohort. **(E)** The expression features of six key TRGs between the low- and high-risk groups in the training cohort.

The flow chart of this study was shown in Figure 1. A total of 50 TRGs were obtained from MSigDB database. The heatmap showed the transcriptional activity of 50 TRGs between LGG patients from the TCGA-LGG dataset and normal tissues from the GTEx dataset (Figure 2A). According to the criteria, 46 genes were identified as differential expression genes, in which 40 genes were upregulated and six genes were downregulated in patients with LGG. Furthermore, univariate Cox regression analysis showed that 34 out of 50 TRGs were related to the prognosis of LGG patients (Figure 2B). Therefore, the 32 genes overlapped between 46 DE-TRGs and 34 prognosis-related TRGs were used for the subsequent analysis (Figure 2C). After that, we conducted the LASSO regression analysis of the filtered 32 genes. Consequently, six candidate genes with non-zero regression coefficients were retained as the key TRGs in LGG (Figures 2D, E). Then, the key TRGs were utilized to construct a TRG-based prognostic risk model. The formula was as follows: Risk model = 0.0475*IL4I1 + 0.0946*STAT1 + 0.0314*SLC36A4 + 0.0451* MAOB + 0.4665*AOX1-0.1485*ALDH2 (Figure 3A). And the training cohort was fully classified into the low- and high-risk groups according to the median value of risk scores. As shown in Kaplan-Meier plot, the patients in high-risk group experienced an obviously worse OS than the patients in low-risk group (HR = 4.07, 95% CI = 2.86–5.80, *P* < 0.001, Figure 3B). And the AUC values based on the time-dependent ROC curve were 0.869, 0.835, and 0.733 at 1-, 3-, and 5-year, indicating the accurate prognostic prediction of the risk model (Figure 3C). In addition, we compared the AUC values of risk model constructed based on key TRGs with those of similar models (Lai et al., 2022a; Zhang et al., 2022; Zhao et al., 2022). And the results showed that our model have the prognostic predictive power with a moderate robustness (Supplementary Figure 1). As shown in Figure 3D, the risk plots demonstrated that the mortality rates of LGG patients elevated as the risk scores increased. And the heatmap suggested the expression features of the key TRGs in our risk model between the low- and high-risk groups in the training cohort (Figure 3E).

#### 3.1.2. Construction of a nomogram based on the risk score and clinicopathological factors

**FIGURE 4:**
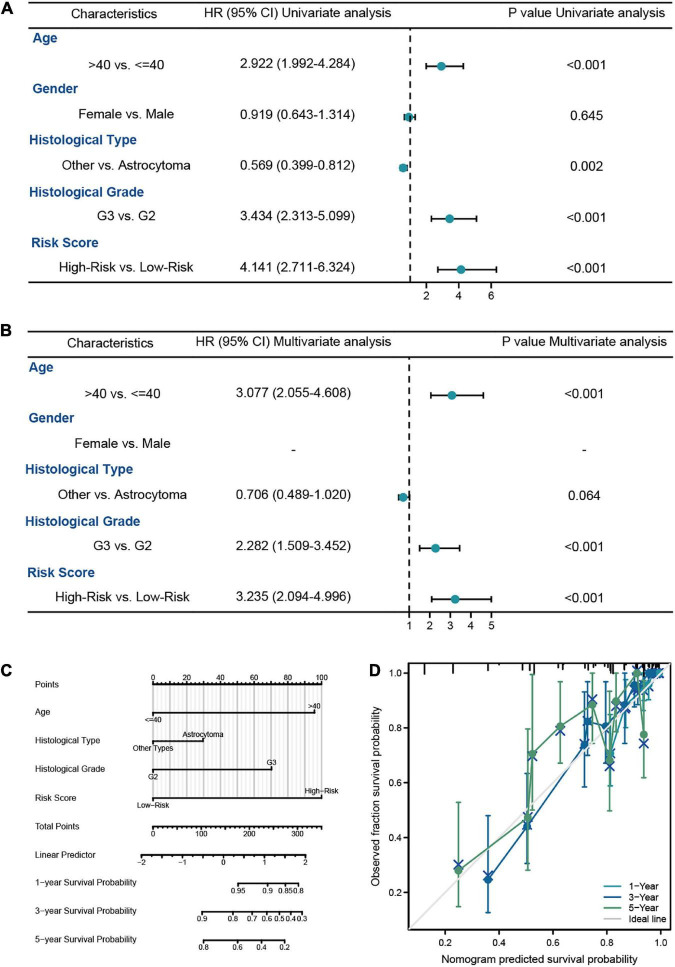
The survival analysis of risk model in LGG patients from TCGA-LGG dataset. **(A,B)** The univariate and multivariate Cox regression analysis of OS in LGG patients from the training cohort. **(C)** The nomogram for the prediction of 1-, 3-, and 5-year OS in LGG patients. **(D)** Calibration plot of the nomogram in LGG patients.

In this analysis, we constructed the univariate and multivariate Cox regression analysis to identity the independent prognostic predictors in LGG patients. The results showed that the age of patients (HR = 3.306, 95% CI = 1.919–5.693, *P* < 0.001), histological grade (HR = 2.951, 95% CI = 1.614–5.395, *P* < 0.001), IDH1 status (HR = 0.401, 95% CI = 0.224–0.717, *P* = 0.002), and risk score (HR = 2.973, 95% CI = 1.655–5.338, *P* < 0.001) were the independent prognostic predictors of the OS in LGG patients (Figures 4A, B). Then we used the independent predictors mentioned above to establish the nomogram for the prediction of 1-, 3-, and 5-year OS in LGG patients with the *C*-index = 0.827 (0.802–0.852), which indicated that the nomogram was the ideal model for predicting the prognosis of LGG patients (Figures 4C, D).

#### 3.1.3. Correlation analysis of the risk score with clinicopathological factors

To investigate the relationship between the risk model and the clinicopathologic characteristics of LGG patients, we explored the distribution of different clinicopathological factors in the low- and high-risk groups (Supplementary Figure 2A). And we found the age and histological grade were significant positive correlated with the risk score. In addition, we observed that astrocytoma 1p/19q non-codel and IDH1 wild type subgroups had higher risk score, which were the poor prognostic factors in LGG patients (Supplementary Figures 2B−G).

#### 3.1.4. Evaluation of the tumor immune characteristics between low- and high-risk groups

**FIGURE 5:**
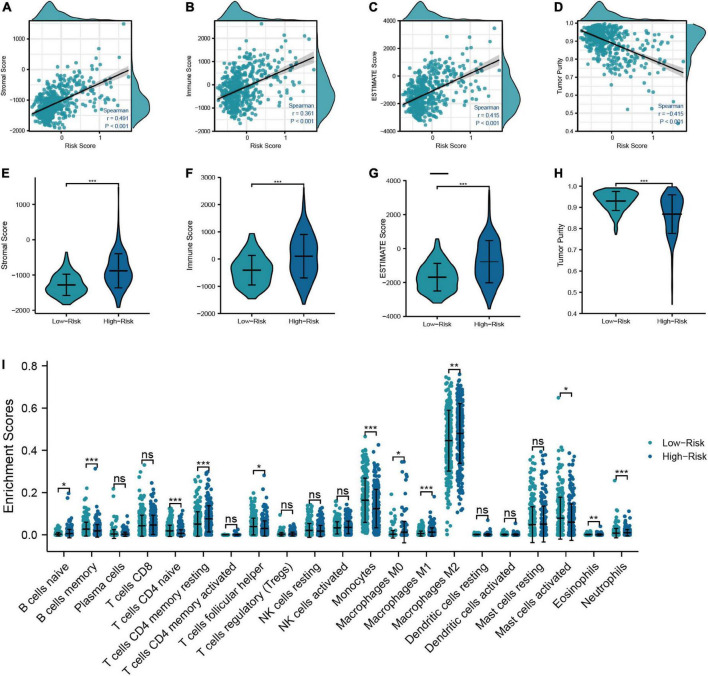
Relationship between the risk model and TIME. **(A–D)** Correlation analysis of risk score with tumor microenvironment scores (Stromal score, immune score, ESTIMATE score, and tumor purity). **(E–H)** Comparison of tumor microenvironment scores between low- and high-risk groups. **(I)** Differences in the infiltration level of immune cells between risk groups (***P < 0.001, **P < 0.01, *P < 0.05).

In order to examine the correlation between the risk model and the TIME, we calculated the immune score, stromal score, ESTIMATE score, and tumor purity of LGG patients. The results showed that the immune score, stromal score, and ESTIMATE score had the positive correlations with the risk score, which means that they were higher in high-risk patients than in low-risk patients (Figures 5A−H). We next used the “CIBERSORT” analysis to investigate the relative proportions of 22 immune cells. As demonstrated in Figure 5I, 12 immune cells had different infiltration between low- and high-risk patients. And the infiltration of naive B-cells, T-cells resting CD4 memory, M0, M1, M2 macrophages, and neutrophils was greater in the high-risk group, which signified that the immune infiltration may play a vital role in the poor prognostic for LGG patients. Finally, we further specifically explored the correlation of risk scores with markers of macrophage and neutrophil in LGG tissues to validate the results of the previous immune infiltration analysis. As shown in Supplementary Figure 3, the expression levels of markers of neutrophil and macrophage were positively correlated with risk scores, in accordance with the results of immune infiltration analysis.

### 3.2. Construction of molecular subtypes based on TRGs in LGG

#### 3.2.1. Consensus clustering and the construction of molecular subtypes based on the key TRGs

**FIGURE 6:**
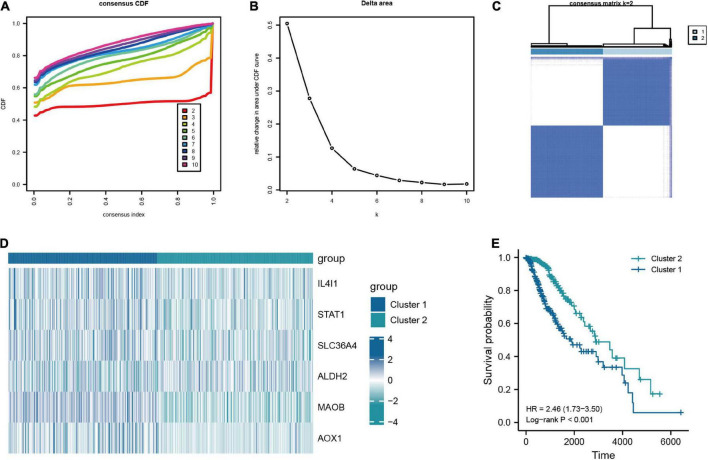
Consensus clustering analysis based on the key TRGs in LGG. **(A)** CDF curve distribution for consensus clustering. **(B)** Relative change in area under CDF curve when *k* = 2–10. **(C)** Unsupervised clustering analysis based on the key TRGs. **(D)** Expression distribution of six key TRGs in cluster 1 and cluster 2. **(E)** The Kaplan-Meier plot comparing the cluster 1 and cluster 2 in OS.

In addition, to further investigate the characteristic of TRGs in LGG, we exploited the novel molecular subtype. Firstly, six TRGs were selected for clustering. We found that the highest intra-cluster correlation was obtained when *K* = 2 by increasing the clustering variable (*K*) from 2 to 10 (Figure 6C and Supplementary Figure 4). PCA revealed that LGG patients in TCGA cohort could be separated remarkably into two clusters according to expression profiles of the six TRGs (Supplementary Figure 5A). This meant that the six TRGs could effectively divide the LGG patients from the training cohort into two clusters. Thus, the molecular subtypes of two clusters can be precisely defined at *K* = 2. Figures 6A, B represented the CDF of the consistent clustering and the relative change in the area under the CDF curve when *K* is different values, respectively. The six TRGs expression profiles between the two clusters were presented in a heatmap (Figure 6D). Except for ALDH2, the expression levels of other TRGs were upregulated in cluster 1. The Kaplan-Meier survival curve based two clusters showed that the prognostic of cluster 1 was worse than that of cluster 2 (HR = 2.46, 95% CI = 1.73–3.50, *P* < 0.001, Figure 6E). In addition, Supplementary Figure 5B demonstrated the GSEA results of cluster 1 and cluster 2 patients with LGG, and we found that cluster 2 were enriched in interactions between immune cells and microRNAs in tumor microenvironment pathway, PD-1 signaling pathway, MET promotes cell motility pathway, neutrophil degranulation pathway, and tryptophan metabolism pathway.

#### 3.2.2. Construction of a nomogram based on molecular subtypes and clinicopathological factors

**FIGURE 7:**
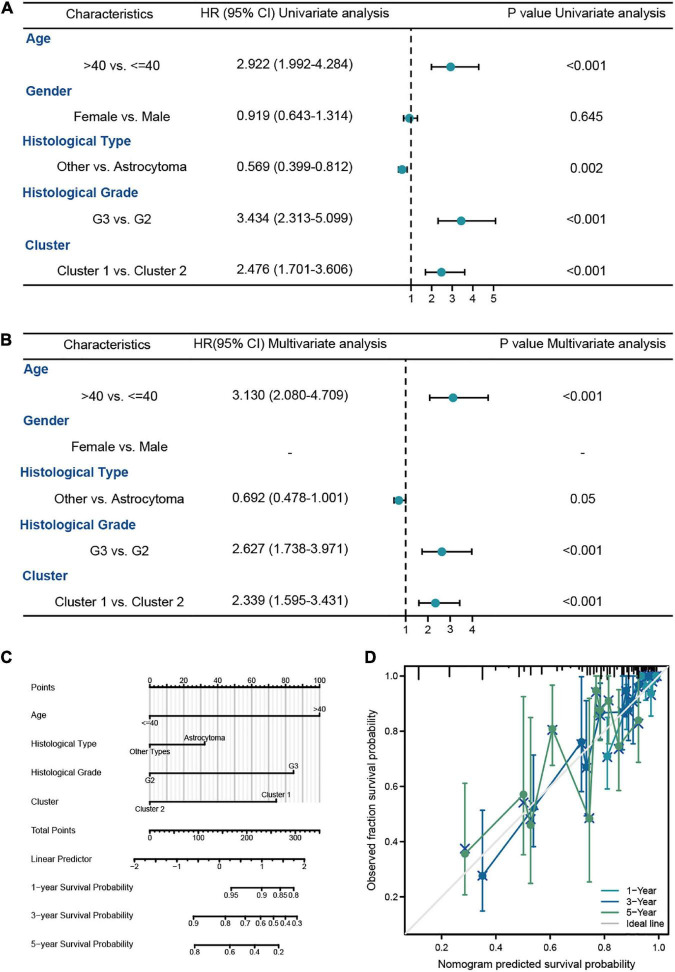
The survival analysis of molecular subtypes in LGG patients from TCGA-LGG dataset. **(A,B)** The univariate and multivariate Cox regression analysis of OS in LGG patients from the training cohort. **(C)** The nomogram for the prediction of 1-, 3-, and 5-year OS in LGG patients. **(D)** Calibration plot of the nomogram in LGG patients.

In order to explore the prognostic value of molecular subtype in LGG patients, the univariate and multivariate Cox regression analysis was carried to perform the survival analysis based on the clinical data from the training cohort (Figures 7A, B). It could be seen from Figure 7B that the age of patients, histological grade, 1p/19q codeletion, IDH1 status, and clusters were related to OS of LGG patients. Excepted 1p/19q codeletion, age (HR = 3.478, 95% CI = 2.034–5.947, *P* = 0.074), histological grade (HR = 3.487, 95% CI = 1.916–6.347, *P* < 0.001), IDH1 status (HR = 0.319, 95% CI = 0.183–0.556, *P* < 0.001), and clusters (HR = 1.787, 95% CI = 1.063–3.001, *P* = 0.028) were all independent predictors of prognosis in LGG patients. Furthermore, the independent predictors mentioned above were employed to construct the nomogram of 1-, 3-, and 5-year OS of LGG patients from the training cohort with the C-index of predicted OS 0.79 (0.769–0.811), suggesting that the nomogram had the superb ability to predict the prognosis of LGG patients (Figures 7C, D).

#### 3.2.3. Correlation analysis of molecular subtypes with clinicopathological factors

The connections between clinicopathologic factors and the molecular subtype were presented in a Sankey diagram (Supplementary Figure 6A). There were significant differences in the distribution of age, histological type, histological grade, 1p/19q codeletion, and IDH1 status between cluster 1 and cluster 2, while the distribution of gender between the two groups did not have statistically significant differences (Supplementary Figures 6B−G).

#### 3.2.4. Analysis of immunological features based on molecular subtypes

**FIGURE 8:**
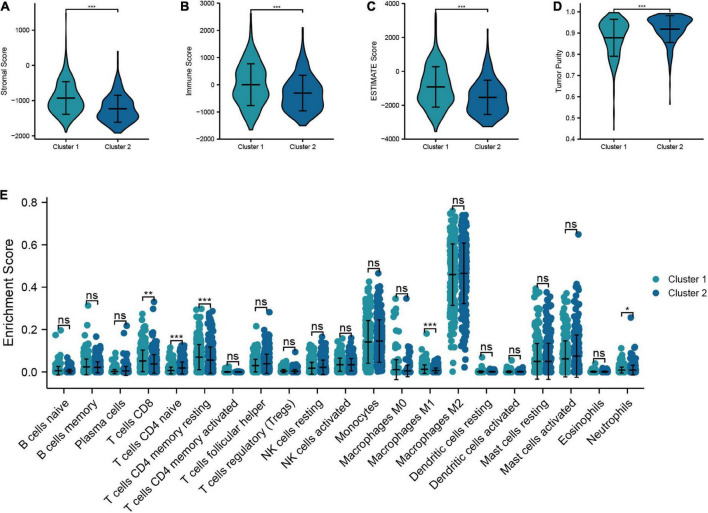
Relationship between the molecular subtypes and TIME. **(A–D)** Comparison of tumor microenvironment scores between cluster 1 and cluster 2 (Stromal score, immune score, ESTIMATE score, and tumor purity). **(E)** Differences in the infiltration level of immune cells between two clusters (***P < 0.001, **P < 0.01, *P < 0.05).

In this section, we analyzed the discrepancy in TIME between cluster 1 and cluster 2. The results suggested that cluster 1 had higher stromal score, immune score, and ESTIMATE score than cluster 2, while the tumor purity in cluster 1 was lower than that in cluster 2 (Figures 8A−D). In addition, the “CIBERSORT” algorithm was utilized to calculate the distinction of immune cell infiltration between cluster 1 and cluster 2, and the outcomes demonstrated a significant discrepancy in the content of immune cells between the two groups. The level of infiltration of T-cells CD8, T-cells CD4 memory resting, M1 macrophages, and neutrophils in cluster 1 were higher than that of cluster 2, while cluster 2 had a higher proportion of T-cells CD4 naive infiltrating compared to cluster 1 (Figure 8E). Overall, these results confirmed that TRGs strongly affect the TIME in LGG patients.

### 3.3. External validation of the TRGs prognostic risk model and molecular subtypes

We chose the CGGA dataset as the validation set to further verify the predictive prognostic performance of the TRGs signature. The outcomes were shown in Supplementary Figures 7A, B were the survival time and survival state scatter diagram and the TRGs expression heatmap between the low- and high-risk groups in the validation cohort, respectively. The Kaplan-Meier analysis of low- and high-risk groups indicated a significant difference in the survival probability, and high-risk group experienced an obviously worse OS than low-risk group (HR = 3.16, 95% CI = 2.18–4.60, *P* < 0.001, Supplementary Figure 7C). The ROC curve showed that the TRGs signature had excellent predictive efficacy for 1-year (AUC = 0.713), 3-year (AUC = 0.791), and 5-year (AUC = 0.735) survival (Supplementary Figure 7D). In addition, we verified the molecular subtype in the validation cohort. The results showed that when LGG patients from the validation cohort were divided into two clusters, the intra-cluster correlation was highest, which was in line with the findings described in the validation cohort (Supplementary Figure 7E). Supplementary Figure 7F showed the TRGs expression levels between two clusters in the validation cohort. The Kaplan-Meier curve demonstrated that the OS in cluster 1 is shorter than that in cluster 2 (Supplementary Figure 7G).

### 3.4. Expression, prognostic value, and TIME analysis of TRGs

**FIGURE 9:**
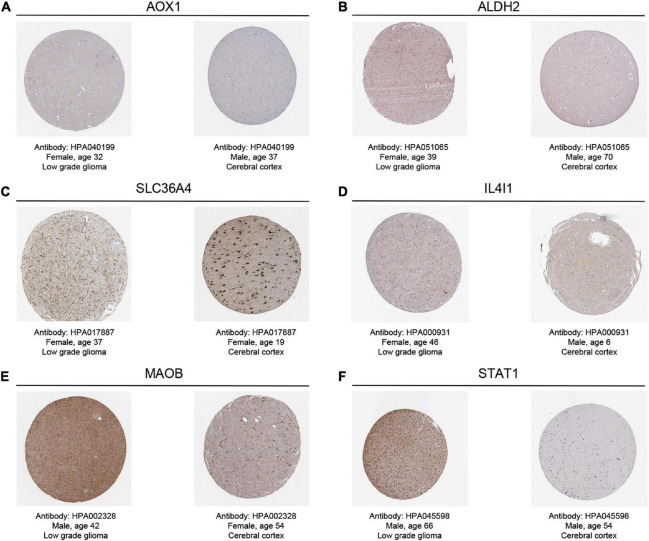
The representative immunohistochemistry images for the key TRGs of LGGs and normal tissues in the HPA dataset. **(A)** AOX1, **(B)** ALDH2, **(C)** SLC36A4, **(D)** IL4I1, **(E)** MAOB, and **(F)** STAT1.

By searching in HPA database, we investigated the expression of six TRGs at the protein level. The representative immunohistochemistry staining images retrieved from the HPA database reflected the protein expression level of the key TRGs in LGG patients (Figure 9). We next explored the prognostic value of six TRGs involved in our study. The Kaplan-Meier analysis indicated that LGG patients with high expression of the IL4I1 (HR = 2.28, 95% CI = 1.57–3.31, *P* < 0.001), STAT1 (HR = 2.39, 95% CI = 1.53–3.74, *P* < 0.001), SLC36A4 (HR = 1.82, 95% CI = 1.24–2.67, *P* = 0.001), MAOB (HR = 3.17, 95% CI = 2.01–5.00, *P* < 0.001), and AOX1 (HR = 1.86, 95% CI = 1.28–2.70, *P* = 0.003) experienced a poor prognosis in the CGGA dataset, while patients with high expression of the ALDH2 (HR = 0.35, 95% CI = 0.23–0.53, *P* < 0.001) had a better OS (Supplementary Figure 8). Subsequently, Spearman correlation analysis was performed to evaluate the correlation between the six TRGs and stromal score, immune score, ESTIMATE score, and tumor purity (Supplementary Figure 9A). The outcomes showed that IL4I1 had the strongest positive correlation with the stromal score and the strongest negative correlation with the tumor purity (Supplementary Figures 9B, C). Finally, the “CIBERSORT” analysis was used to calculate the infiltration level of 22 immune cells, and the correlation analysis showed a significant positive relationship between STAT1 and M1 macrophages and a significant negative relationship between IL4I1 and T-cells follicular helper (Supplementary Figure 9D). Taken together, the above results confirmed that TRGs strongly affect the immune infiltration in patients with LGG.

## 4. Discussion

Glioma is one of the most common primary malignant tumors in CNS (Yang et al., 2022). In recent years, there have been significant progresses regarding the standard treatment of LGG, including surgical resection, targeted radiation, and chemotherapy (van den Bent et al., 2018; Wang and Mehta, 2019). However, some patients with LGG still have a certain rate of recurrence and malignant transformation after standardized treatment, which can rapidly develop into highly aggressive secondary glioblastoma with a poor survival prognosis (Xu et al., 2020). Therefore, it is of great clinical importance to find an accurate biomarker to establish the relevant molecular subtypes of LGG, as well as to predict the prognosis of LGG patients, in order to support the more precise and individualized treatment. In recent years, the relationship between tryptophan metabolism and tumors has been the focus of the research. Many studies have revealed that tryptophan metabolism is participated in the pathogenesis of tumors, especially gliomas, through multiple mechanisms (Kim and Tomek, 2021; Platten et al., 2021). However, there is still a paucity of studies on the combined effect of TRGs in LGG patients. Therefore, starting from the differential expression of TRGs in LGG, we constructed a stable and effective predictive risk model as well as established two different molecular subtypes, then explored their relationship with clinicopathological factors and TIME, so as to support more precise and individualized treatment for patients with LGG.

In this study, we first explored the expression characteristics of 50 TRGs in the TCGA-LGG dataset and identified 46 DE-TRGs, of which 40 genes were upregulated and six genes were downregulated in LGG patients. Additionally, the results of the survival analysis on TRGs showed that the expression levels of 34 genes were significantly correlated with the OS of LGG patients. By overlapping the DE-TRGs with the prognostic TRGs, we acquired 32 prognostic DE-TRGs. The LASSO regression analysis was performed shortly after, and six key TRGs (IL4I1, STAT1, SLC36A4, ALDH2, MAOB, and AOX1) were identified. Next, we constructed a risk model containing six key TRGs to predict the prognosis of LGG patients, thus dividing LGG patients into the low- and high-risk group. The patients in the high-risk group differed significantly from those in the low-risk group in terms of prognosis, clinicopathological factors, and immune infiltration. Multivariate Cox regression analysis suggested that the risk model could be considered as an independent risk factor for predicting the prognosis of LGG patients. Subsequently, we established a nomogram containing risk scores and clinicopathological factors in order to more accurately predict the prognosis of LGG patients and to provide new perspectives and ideas for targeted therapy. Moreover, we divided LGG patients into two molecular subtypes based on the expression of six key TRGs. The Kaplan-Meier survival analysis showed that OS was significantly shorter in cluster 1 than in cluster 2. There were also remarkable differences in the aggregation of molecular pathways, clinicopathological factors as well as TIME between the two clusters. Then we constructed a nomogram on the basis of molecular subtypes and clinicopathological factors to further enhance the predictive prognostic performance of molecular subtypes. Finally, we have successfully validated the risk model and molecular subtype through external validation, demonstrating their robust prognostic value. Our study is crucial for the development of effective molecular biomarkers to improve the clinical management and to reduce the mortality in LGG patients.

Accumulating evidence have demonstrated that tryptophan metabolic reprogramming is closely associated with the process of tumor development (Platten et al., 2021). In this study, we determined six TRGs that could serve as key genes for the prognosis of LGG, including: IL4I1, STAT1, SLC36A4, ALDH2, MAOB, and AOX1. Studies have shown that the overexpression of IL4I1 is related to poor survival in glioma patients (Sadik et al., 2020). It plays an essential role in tumor development mainly by affecting tryptophan catabolism, which is mediated to produce I3P, and it could lead to the production of indole metabolites and kynurenine (KYN). The latter serves as a ligand to activate the transcription factor AHR, which may enhance tumor progression by promoting cancer cell movement (Sadik et al., 2020), inhibiting T-cells activation and proliferation (Boulland et al., 2007), and regulating the development and function of B-cells (Aubatin et al., 2018). Signal transducer and activator of transcription 1 (STAT1) is a transcriptional active factor that has been previously studied to promote the progression of many tumors (Greenwood et al., 2012; Arzt et al., 2014). It plays a pivotal role in indoleamine 2,3-dioxygenase 1 (IDO1) expression (Jeong et al., 2009). IDO1 is the rate-limiting step that catalyzes tryptophan catabolism and mediates the conversion of tryptophan to KYN metabolite (Blair et al., 2019). IDO1 metabolism can deplete the tryptophan from the tumor microenvironment, leading to the impairment of T-cells activation, thereby achieving immunosuppression of the tumor microenvironment and escaping attack by the host immune system (Terness et al., 2002). Solute carrier family 36 member 4 (SLC36A4) is an amino acid transporter protein with high affinity for tryptophan (Pillai and Meredith, 2011). Previous studies have found that tumor cells can transport the tryptophan metabolite KYN into cytotoxic CD8^+^ T-cells by mediating SLC36A4. Then PD-1 in the nucleus of the T-cells is upregulated by the activation of AHR within CD8^+^ T-cells, thereby promoting tumor evasion of immune monitoring and tumor progression (Murray et al., 2014; Liu et al., 2018). Aldehyde dehydrogenase 2 (ALDH2) is a mitochondrial enzyme participating in various physiopathological processes (Chen C. H. et al., 2014), including catalyzing indole-3-acetaldehyde to indole in tryptophan metabolism. Various researches have found that ALDH2 may play an important part in cancer development and progression (Moreb et al., 2012; Andrew et al., 2015). Patients with high ALDH2 expression in hepatocellular carcinoma have a good prognosis (Zhang and Fu, 2021), and the inhibition of ALDH2 expression could enhance tumor cell proliferation, stemness and migration, resulting in poor prognosis in lung adenocarcinoma patients (Li et al., 2019), which is consistent with our findings. Monoamine oxidase B (MAOB) is an enzyme responsible for the metabolism of serotonin and plays an essential part in the degradation of the tryptophan metabolite 5-HT (Sadkowski et al., 2013; Jin et al., 2015). Compared to normal tissues, MAOB is reduced in some types of cancer, while elevated in other specific types (Chen P. H. et al., 2014; Hodorová et al., 2018). Zhang et al. (2019) revealed that MAOB expression was lower in endometrial cancer tissues than in normal tissues and that downregulation of MAOB was associated with poorer survival, which is consistent with our study. In contrast, Sharpe and Baskin (2016) found that the expression level of MAOB was upregulated in gliomas, and that its expression level was positively correlated with the pathological grade. Aldehyde oxidase 1 (AOX1),a flavin-containing monooxygenase, is one of the key enzymes in the tryptophan catabolic process. It has been found that deletion of AOX1 may lead to the accumulation of the tryptophan metabolites kynurenine and nicotinamide adenine dinucleotide phosphate (NADP) (Vantaku et al., 2020). AOX1 has been reported to show a low expression level in breast cancer, prostate cancer, colon cancer, and glioma cancers (Oster et al., 2011; Haldrup et al., 2013; Stavrinou et al., 2015; Ozturk et al., 2016; Vantaku et al., 2020), which are the same as our findings. Meanwhile, Stavrinou et al. (2015) found that the higher WHO grade in astrocytomas was associated with a decrease in intensity of AOX1 expression. Interestingly, in the O6-benzylguanine protocol applied to patients with recurrent malignant glioma, the lower the expression of AOX1 expression in patients, the more effective the treatment effect and the lower the resistance to drugs, which in turn represents a better prognosis for the patients with glioma (Quinn et al., 2009). Nevertheless, our study showed that AOX1 is a risk factor for the prognosis of LGG patients. In summary, our study identified the predictive power of TRGs for the prognosis of LGG patients. Further experiments, however, are still needed to clarify the actual clinical significance of these TRGs prognostic biomarkers.

In this study, LGG patients were divided into a low-risk group and a high-risk group by the risk model. Survival analysis showed significant differences in prognosis between the two groups. The TRGs risk model and the nomogram integrating clinicopathological factors and IDH1 status of LGG patients could independently predict the prognosis of LGG patients with a strong generalization ability. They can systematically obtain accurate and robust results in predicting the prognosis of LGG patients, helping to more precisely assess the clinical outcomes of LGG patients and provide a more personalized prognostic evaluation strategy for LGG patients. Although the results of our analysis showed that the risk score constructed based on TRGs was an independent prognostic factor for LGG patients. However, by comparison with similar risk models (Lai et al., 2022a; Zhang et al., 2022; Zhao et al., 2022), our risk model may not be the optimal predictive model for LGG patients. In contrast, the time-dependent ROC curves showed that the AUC values of the risk model constructed based on TRGs were all above 0.7 at 1-, 3-, and 5-year OS in LGG patients, which suggested that our prognostic model had a moderate robustness. Tumor immunotherapy has been a hot topic in the field of oncology, such as targeting metabolism to improve the tumor microenvironment (Suarez-Carmona et al., 2017). The KYN can activate AHR, which inhibit the anti-tumor immunity. Therefore, tumor immunity is a crucial event in LGG involving tryptophan metabolism (Hinshaw and Shevde, 2019). We analyzed the differences in immune infiltration between low- and high-risk groups to further elucidate the mechanisms of prognostic differences between risk groups. The results of the ESTIMATE-immune analysis suggested that patients in the high-risk group had higher immune scores, suggesting a dysregulation of TIME and abnormal aggregation of immune cells in the high-risk group. Then we used the “CIBERSORT” algorithm to compare the differences in immune cell infiltration between the low- and high-risk groups and found that the high-risk group had higher levels of macrophage and neutrophil aggregation. Moreover, we found that the expression levels of markers of neutrophil and macrophage were positively correlated with risk scores. The correlation between each TRGs and immune infiltration was further analyzed, and the findings revealed the highest correlation between IL4I1 and immune score, and that AOX1, IL4I1, MAOB, and STAT1 all had a positive correlation with macrophage infiltration. Glioma-associated microglia and macrophages are abundant in glioma, accounting for approximately 40% of tumor tissues (Delwar et al., 2018). GBM loses its normal immune function during tumor progression and could produce a variety of cytokines and angiogenic factors, which is one of the important factors for a poor prognosis of glioma (Voisin et al., 2010). It has been found that the immune function of glioma-associated microglia and macrophages will be influenced by the surrounding tumor tissue when it is recruited to the TIME and transformed from a pro-inflammatory phenotype to an anti-inflammatory phenotype, which acts as a facilitator for the development of glioma (Gu et al., 2017; Henrik Heiland et al., 2019). In addition, the number of circulating and infiltrating neutrophils are correlated with the pathological grade of glioma. In preclinical glioma models, neutrophils promoted tumor growth and the number of neutrophils infiltrating was associated with the resistance to treatment of acquired anti-vascular endothelial growth factor. In the circulation, neutrophils promote tumor growth by producing arginase I to induce immunosuppression; in tumor areas, neutrophils secrete elastase, which assists in glioma infiltration and tumor cell proliferation (Rahbar et al., 2016; Massara et al., 2017). It is thus clear that tryptophan metabolism plays a key role in tumor promotion and immunosuppression in LGG.

With the gradual improvement of tumor pathology diagnosis and refinement to the molecular subtype stage, the research of precision medicine and the promotion of accurate diagnosis and treatment will benefit tumor patients. The molecular subtype can be used to predict the prognosis of patients with glioma and direct clinical treatment. Glioma is a highly heterogeneous tumor, and the molecular genetics vary greatly within gliomas with similar histologic features, leading to a large discrepancy in prognosis among individuals with the same WHO pathologic histologic classification (Berghoff et al., 2017). LGG patients can be classified into different molecular subtypes based on gene expression characteristics, and this study has been widely researched nowadays (Xiao et al., 2022). 2016 WHO has included molecular pathology in the pathological diagnosis system and redefined the pathological classification of glioma, which has rapidly changed our recognition (Nabors et al., 2020). Mutations in position 132 of the Isocitrate Dehydrogenase 1 (IDH1) gene exist in more than 80% of LGG, and the patients with IDH1 mutations indicate a better prognosis (Berghoff et al., 2017). The deletion of the short arm of chromosome 1 and the long arm of chromosome 19 (1p/19q) occurs mainly in oligodendrogliomas and, to a few extent, in astrocytomas, which is a hallmark of oligodendrogliomas (Eckel-Passow et al., 2015). According to the study (Berghoff et al., 2017), the prognostic value of IDH mutation status is better than histological grade in the LGG, and the incidence of 1p/19q codeletion in oligodendroglioma is as high as 80–90%. Although IDH mutations are currently the most widely recognized molecular biomarkers for the molecular subtype of LGG, none of the precision therapies targeting IDH mutations have achieved positive therapeutic outcomes (Machida et al., 2020). In our study, TRGs could effectively divide LGG patients into two molecular subtypes with significant differences in terms of prognosis and immune cell infiltration, and were validated in the CGGA dataset. The molecular subtype on the basis of tumor genetics could more accurately determine the clinical prognosis of LGG patients and provide a discriminant basis for tumors that are difficult to diagnose and grade. The establishment of molecular subtype-guided LGG prevention, treatment and drug research is expected to provide both theoretical and practical support for the ultimate realization of the precise diagnosis and treatment strategy for LGG patients.

In this study, we screened TRGs and established a tryptophan-related risk model and molecular subtype based on the six key TRGs, and provided the first comprehensive quantitative assessment on the prognostic value of TRGs in predicting LGG patients, revealing the clinical value of the TRGs risk models and molecular subtypes. Our findings also provide novel insights and perspectives on potential therapeutic strategies and antitumor targets for LGG. Finally, there are some limitations of this study: Firstly, all analyses were based on data from public databases for model construction and validation. Therefore, large-scale prospective studies as well as further *in vivo* and *in vitro* studies to confirm the applicability and stability of the findings of this study are urgently needed to completely investigate the mechanism of tryptophan metabolism in LGG. Secondly, our study found a high infiltration of macrophages and neutrophils in the high-risk group of LGG patients. Also, the markers of macrophages and neutrophils were highly expressed in LGG tissues in the high-risk group. Next, we need to further validate the expression levels of markers of macrophage and neutrophil in the high-risk and low-risk groups by immunohistochemistry. Furthermore, in addition to LASSO, there are also methods such as ridge regression or elastic network regression that can be used for key gene screening (Lai et al., 2022b,c). In this study, we only conducted LASSO regression analysis to screen genes, and we need to further combine multiple statistical methods for screening genes in the future to construct the most consistent prediction model. In addition, the impact of TRGs-based risk model and molecular subtype on immune infiltration and its value in drug screening still need to be further explored. Finally, the current large dataset lacks some important clinical information, such as surgery, chemotherapy modalities, etc., and therefore cannot be included in our study for analysis. Therefore, we need to further clarify the significance of tryptophan metabolism in guiding chemotherapy resistance and immune-related therapy in large prospective clinical trials.

## 5. Conclusion

In summary, we conducted a comprehensive analysis of TRGs, investigated the expression pattern and prognostic value of TRGs in patients with LGG, and constructed a novel risk model and molecular subtypes. Notably, both the risk model and molecular subtypes we developed could accurately predict the prognosis of LGG patients and were further confirmed to be closely related to the clinicopathological factors and TIME of LGG. Our findings suggest new insights and perspectives for exploring potential therapeutic strategies and anti-tumor targets for LGG, and provide theoretical and practical support for the ultimate realization of precision treatment for patients with LGG.

## Data availability statement

The original contributions presented in this study are included in the article/Supplementary material, further inquiries can be directed to the corresponding author.

## Author contributions

WL conceived of the presented idea and designed the research. LL and PD searched databsets and performed the data analysis and statistical analysis. WL, LX, and WY were responsible for writing the manuscript. All authors critically revised the article for important intellectual content and approved the final manuscript.

## Footnotes

1 https://portal.gdc.cancer.gov/, accessed on 1 October 2022

2 https://gtexportal.org/home/, accessed on 1 October 2022

3 http://www.cgga.org.cn/, accessed on 1 October 2022

4 http://www.broad.mit.edu/gsea/msigdb/, accessed on 1 October 2022

5
http://www.proteinatlas.org/
