# Supplementary material for: Identification and validation of a risk model and molecular subtypes based on tryptophan metabolism-related genes to predict the clinical prognosis and tumor immune microenvironment in lower-grade glioma
Source: Front Cell Neurosci. 2023 Feb 28;17:1146686. doi: 10.3389/fncel.2023.1146686 (PMC10011102; doi:10.3389/fncel.2023.1146686)
Supplement: Supplementary file 1 [file Data_Sheet_1.pdf]

Supplementary Material

Identification and validation of a risk model and molecular subtypes based on tryptophan metabolism-related genes to predict the clinical prognosis and tumor immune microenvironment in lower-grade glioma

Wenxia Li, Ling Ling, Lei Xiang, Peng Ding, Wei Yue*

*** Correspondence:** Wei Yue: hhyuewei2008@163.com

1 Supplementary Figures and Tables

1.1 Supplementary Figures

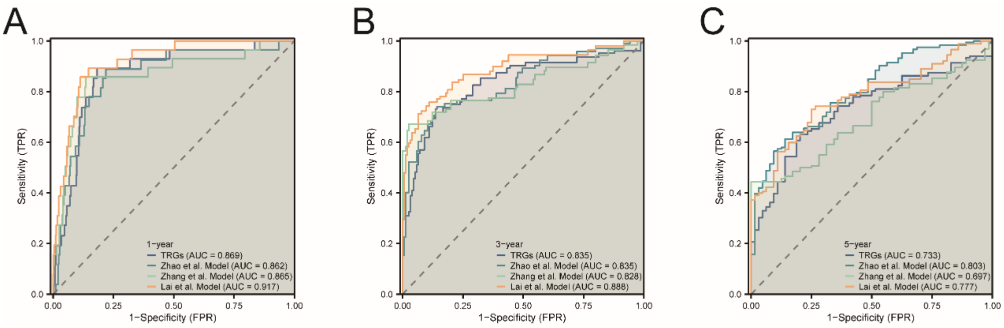

Supplementary Figure. 1. AUC Comparison with three similar risk models in TCGA-LGG database. (A) 1-year time-dependent ROC curves. (B) 3-year time-dependent ROC curves. (C) 5-year time-dependent ROC curves.

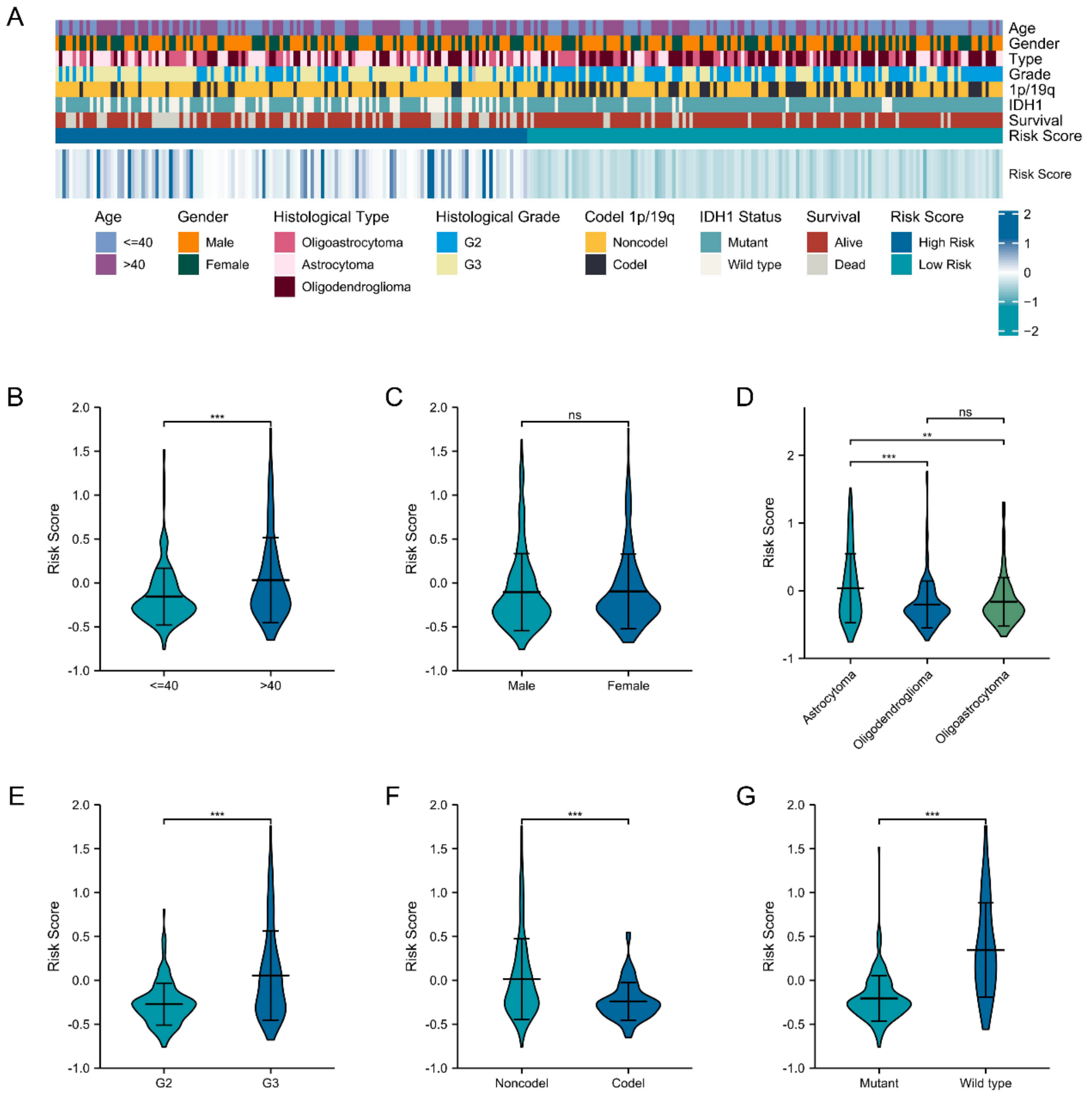

Supplementary Figure. 2. Correlation analysis of risk score with clinicopathological factors. (A) Distribution of clinicopathological factors between low- and high-risk groups. (B-G) Comparison of age, gender, histological type, histological grade, 1p/19q codeletion and IDH1 status of LGG patients between low- and high-risk groups.

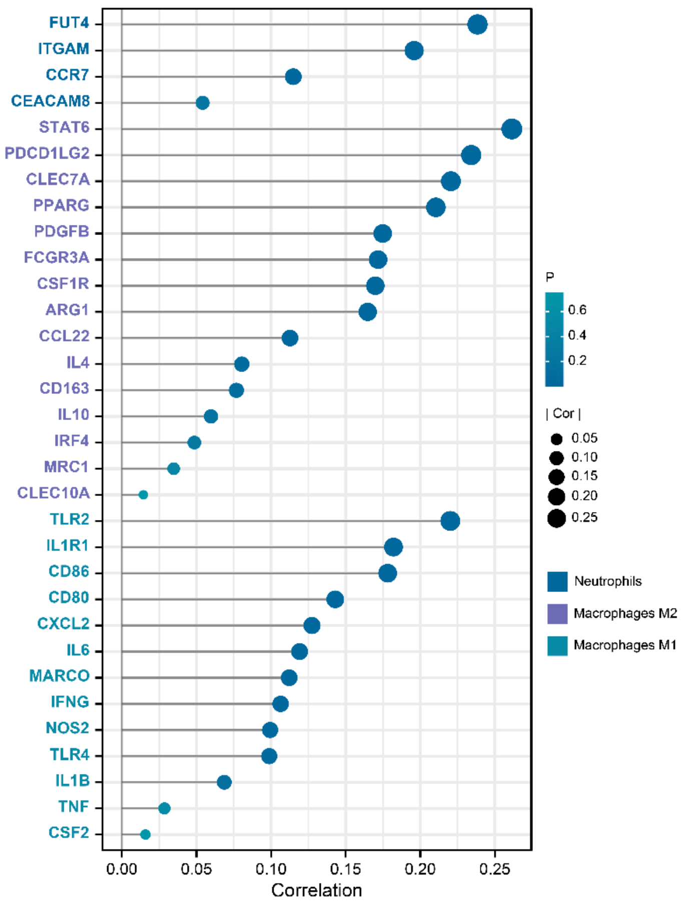

Supplementary Figure. 3. Correlation analysis of risk score with the expression levels of neutrophil and macrophage markers.

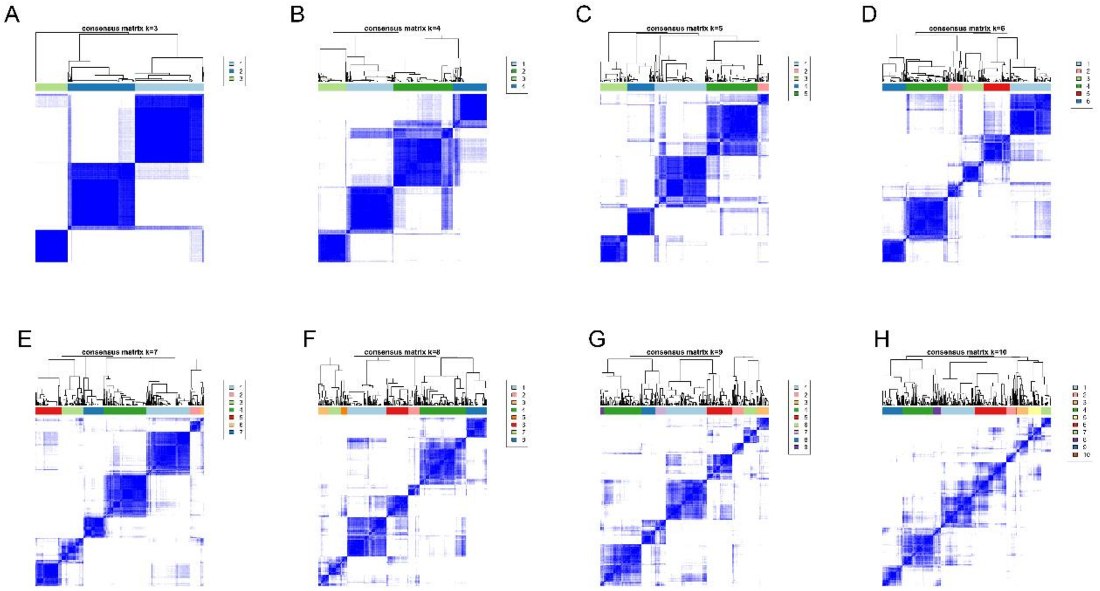

Supplementary Figure. 4. Clustering results when the cluster number K from 2 to 10.

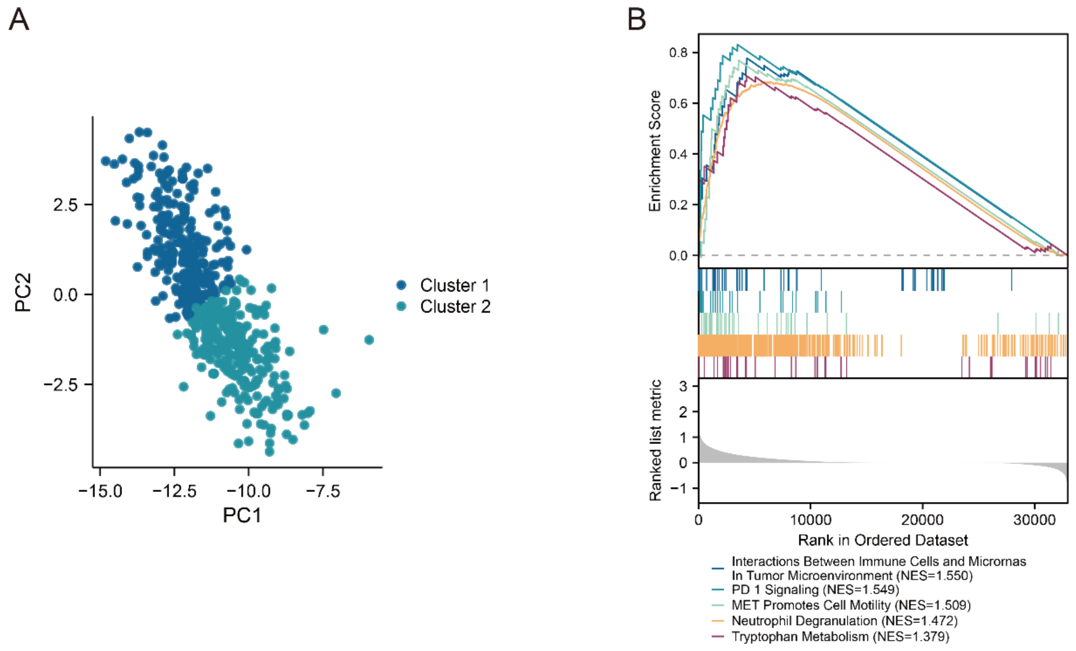

Supplementary Figure. 5. Consensus clustering analysis based on the key TRGs in LGG. (A) PCA plot of LGG patients in cluster 1 and cluster 2 based on the key TRGs. (B) GSEA analysis of cluster 1 and cluster 2 patients with LGG.

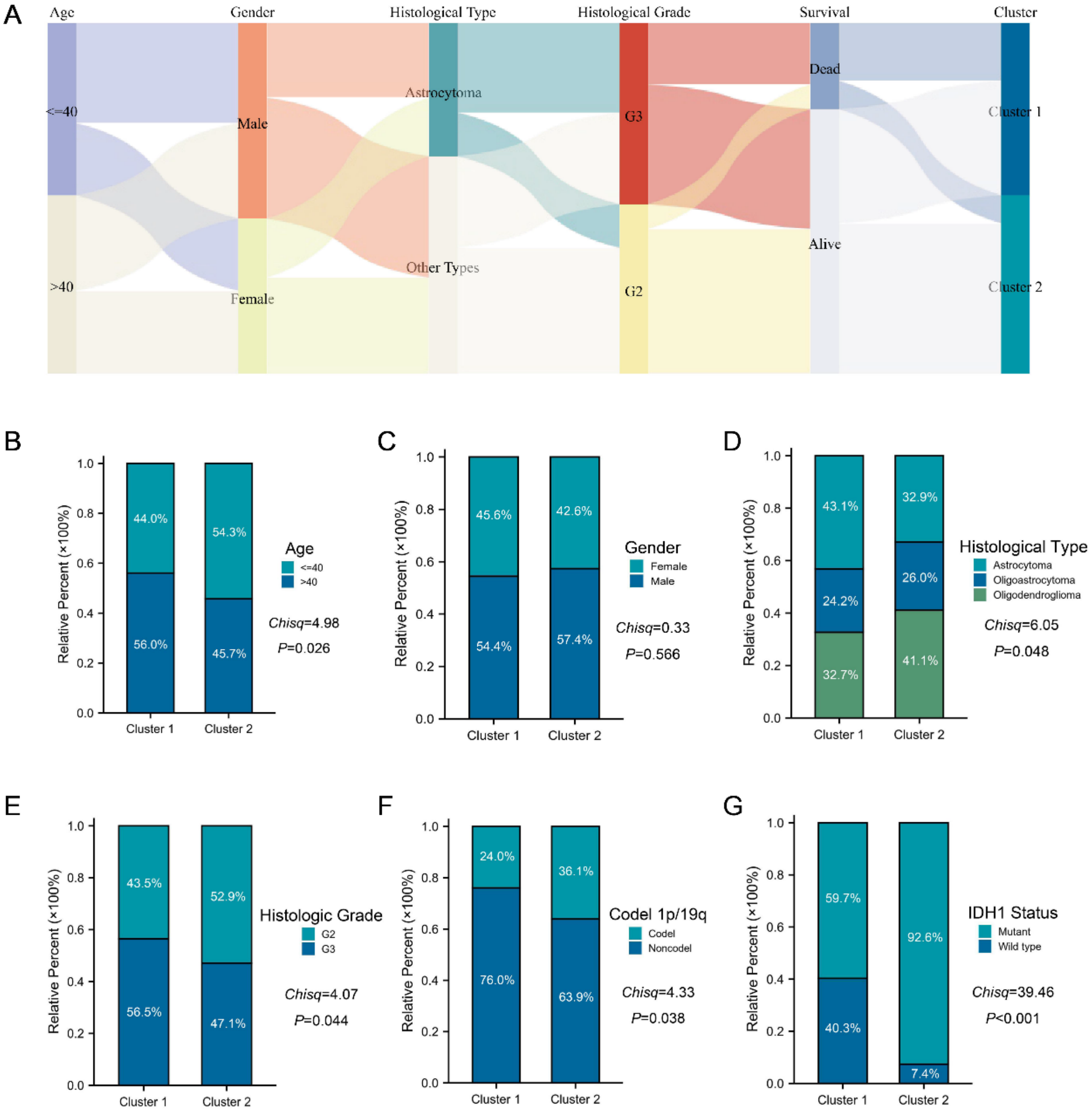

Supplementary Figure. 6. Correlation analysis of clusters with clinicopathological factors. (A) Distribution of clinicopathological factors between cluster 1 and cluter 2. (B-G) Comparison of age, gender, histological type, histological grade, 1p/19q codeletion and IDH1 status of LGG patients between cluster 1 and cluter 2.

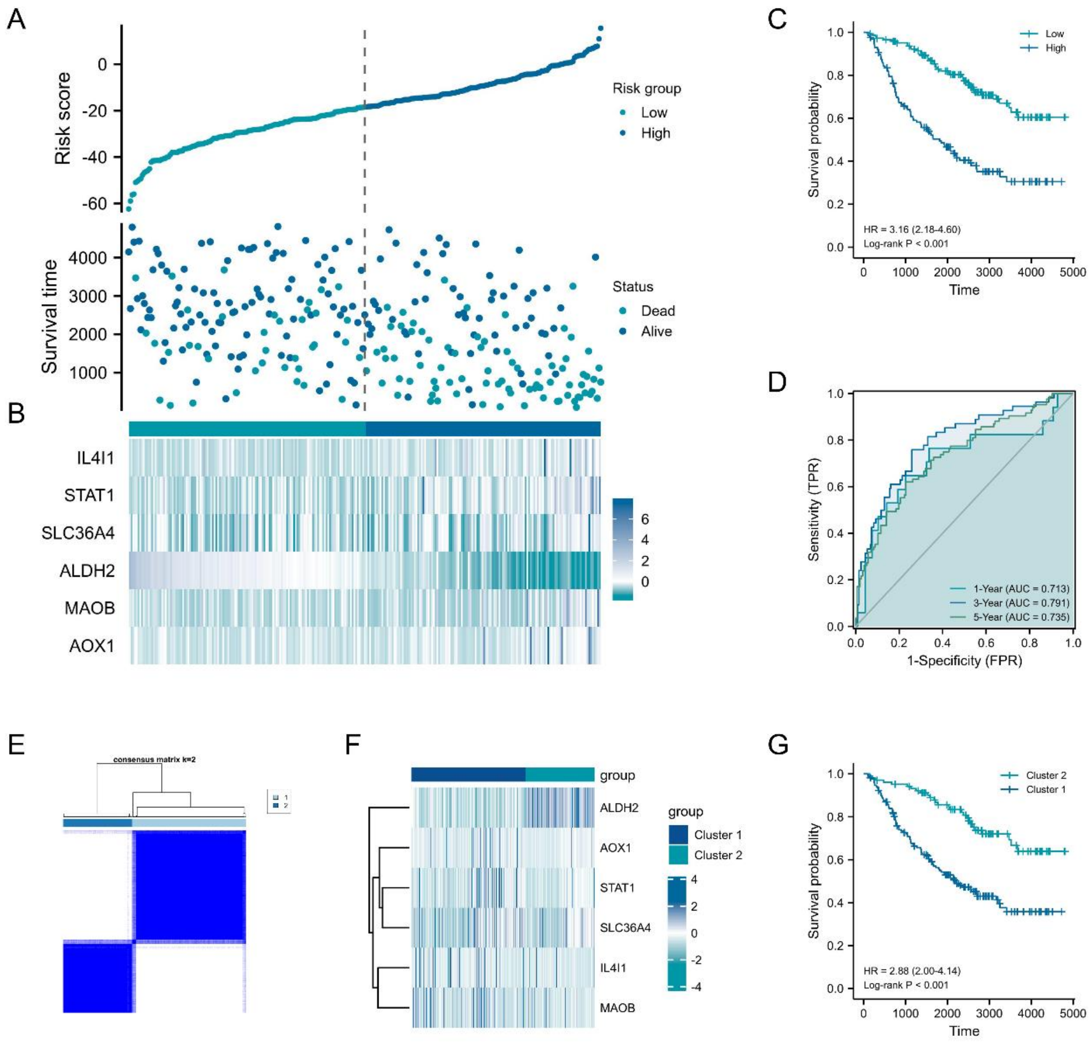

Supplementary Figure. 7. External validation of the TRGs prognostic risk model and molecular subtype in the CGGA dataset. (A) Scatter diagram of risk score and survival status in the validation cohort. (B) The TRGs expression heatmap between the low- and high-risk groups in the validation cohort. (C) The Kaplan-Meier plot comparing the low- and high-risk groups in OS in the validation cohort. (D) The ROC curve for low- and high-risk groups in the validation cohort. (E) Unsupervised clustering analysis based on the key TRGs. (F) Expression distribution of six key TRGs in cluster 1 and cluster 2. (G) The Kaplan-Meier plot comparing the cluster 1 and cluster 2 in OS.

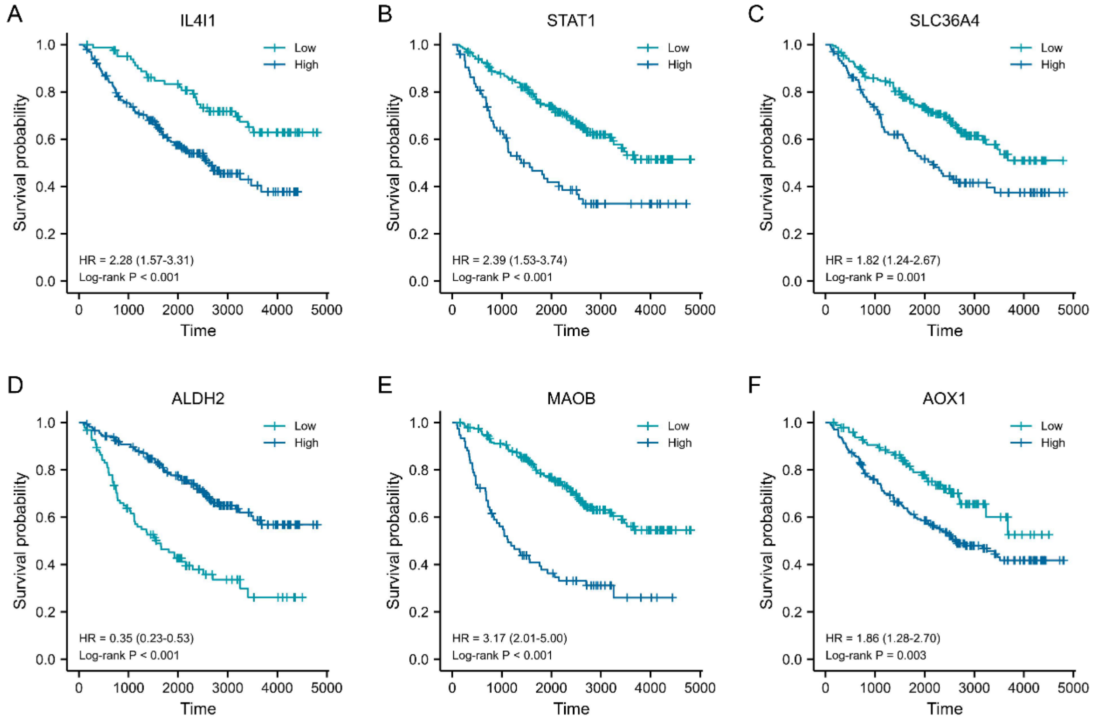

Supplementary Figure. 8. The prognostic value of the key TRGs was explored in the validation cohort, respectively. (A) IL4I1 (B) STAT1 (C) SLC36A4 (D) ALDH2 (E) MAOB (F) AOX1.

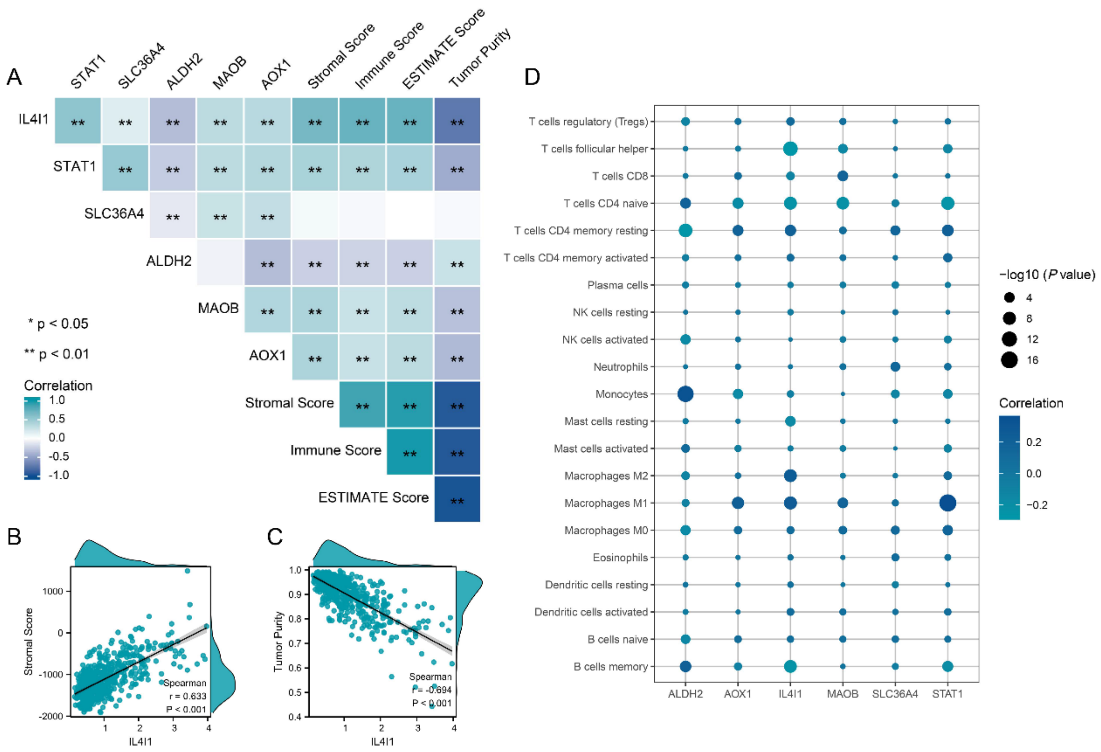

Supplementary Figure. 9. Relationship between the key TRGs and TIME. (A) Correlation analysis of the key TRGs with tumor microenvironment scores (Stromal Score, Immune Score, ESTIMATE Score, and Tumor Purity). (B-C) Correlation analysis of IL4I1 with Stromal Score and Tumor Purity, respectively. (D) Correlation analysis between the key TRGs and the infiltration level of immune cells.

1.2 Supplementary Table

Supplementary Table 1 50 TRGs obtained from the MSigDB database.

Database	Genes
KEGG_TRYPTOPHAN_METABOLISM	WARS2; INMT; TPH; AFMID; ACMSD; AANAT; CYP1A1; CYP1A2; CYP1B1; DDC; IDO2; ECHS1; EHHADH; ALDH2; ALDH1B1; ALDH9A1; ALDH3A2; HAAO; IL4I1; AOC1; GCDH; HADHA; HADH; AOX1; IDO1; ACAT1; ACAT2; MAOA; MAOB; ASMT; OGDH; ALDH7A1; AADAT; OGDHL; TDO2; TPH1; WARS1; CAT; KMO; KYNU
REACTOME_TRYPTOPHAN_CATABOLISM	SLC7A5; AADAT; KYNU; KMO; IDO1; KYAT3; TDO2; ACMSD; HAAO; SLC3A2; KYAT1; SLC36A4; AFMID; IDO2
WP_TRYPTOPHAN_METABOLISM	INMT; TPH2; AFMID; ACMSD; AANAT; CYP1A1; DDC; IDO2; DLD; ECHS1; AHR; ALDH2; HAAO; IL4I1; AOC1; GCDH; GOT2; HADH; AOX1; IDO1; ACAT1; MAOA; ASMT; AADAT; KYAT3; ALDH8A1; STAT1; TDO2; TPH1; CAT; KMO; KYAT1; KYNU
